# Mapping the Antigenicity of the Parasites in *Leishmania donovani* Infection by Proteome Serology

**DOI:** 10.1371/journal.pone.0000040

**Published:** 2006-12-20

**Authors:** Michael Forgber, Rajatava Basu, Kaushik Roychoudhury, Stephan Theinert, Syamal Roy, Shyam Sundar, Peter Walden

**Affiliations:** 1 Department of Dermatology, Venerology and Allergy, Charité - Universitätsmedizin Berlin, Humboldt University Berlin, Germany; 2 Department of Immunology, Indian Institute of Chemical Biology Calcutta, West Bengal, India; 3 Kala-Azar Medical Research Center, Banaras Hindu University Varanasi, Uttar Pradesh, India; Université de Toulouse, France

## Abstract

**Background:**

Leishmaniasis defines a cluster of protozoal diseases with diverse clinical manifestations. The visceral form caused by *Leishmania donovani* is the most severe. So far, no vaccines exist for visceral leishmaniasis despite indications of naturally developing immunity, and sensitive immunodiagnostics are still at early stages of development.

**Methodology/Principle Findings:**

Establishing a proteome-serological methodology, we mapped the antigenicity of the parasites and the specificities of the immune responses in human leishmaniasis. Using 2-dimensional Western blot analyses with sera and parasites isolated from patients in India, we detected immune responses with widely divergent specificities for up to 330 different leishmanial antigens. 68 antigens were assigned to proteins in silver- and fluorochrome-stained gels. The antigenicity of these proteins did not correlate with the expression levels of the proteins. Although some antigens are shared among different parasite isolates, there are extensive differences and no immunodominant antigens, but indications of antigenic drift in the parasites. Six antigens were identified by mass spectrometry.

**Conclusions/Significance:**

Proteomics-based dissection of the serospecificities of leishmaniasis patients provides a comprehensive inventory of the complexity and interindividual heterogeneity of the host-responses to and variations in the antigenicity of the Leishmania parasites. This information can be instrumental in the development of vaccines and new immune monitoring and diagnostic devices.

## Introduction

Leishmaniasis is endemic in 88 countries with approximately 12 million infected and 350 million people at risk (http://www.who.int/en/). The disease is caused by parasites of the genus *Leishmania*, a group of kinetoplastid protozoans, that are transmitted by sandflies as flagellated promastigotes. With the bite of the female vector the parasites are injected into the host to enter and multiply in the phagolysosomes of macrophages as amastigotes [1], [2]. Dependent on the Leishmania species and the immune response of the host, there are three basic clinical manifestations of the disease: cutaneous, mucocutaneous and visceral leishmaniasis (VL) [3], [4]. VL in India, known as Kala azar, is caused by *Leishmania donovani* (LD) and is the most severe form of leishmaniasis. It is characterized by irregular bouts of fever, substantial weight loss, hepatosplenomegaly and anemia. VL inevitably takes a fatal course if not treated. [5]. To worsen matters, in the endemic areas in India treatment is increasingly failing due to resistance of the parasites to the most common anti-leishmanial drug, pentavalent antimony [5]. New drugs such as liposomal amphotericin B and Miltefosin are prohibitively expensive for the most affected populations. These facts and developments stress the urgent need for prophylactic measures and alternative therapies.

Recent studies have revealed that during endemic outbreaks in the endemic areas the prevalence of seropositivity for the leishmanial antigen K39 is significantly higher than the morbidity [6] suggesting that many may have acquired a state of immunity and, in extension, that effective vaccines against VL may be possible. Great efforts are undertaken in search for vaccination strategies to elicit strong, safe and effective immune responses against Leishmania using live or killed parasites [7]–[11], defined subunit vaccines [12]–[14], crude fractions of Leishmania parasites [15] or DNA-vaccination [14], [16]–[21]. The main focus in vaccine development is the elucidation of the range and specificity of anti Leishmania immune responses, and the identification of defined leishmanial antigens. Knowledge of such antigens is required for the development of vaccines. In addition, the antigens can be instrumental in immune monitoring of infection, disease and resistance to disease, and be used in the development of new diagnostics. The specificities of the antibody responses will be determined, on the one side, by the constitution, complexity and variability of the antigenic structures of the parasites and, on the other side, by the immunoglobulin repertoire, and the immune and disease history of the host, and by the specific course of the disease in the individual patients. Given this complexity of the host-parasite-disease relationship, antigenicity and specificity pattern rather than individual serospecificities need to be determined for proper assessment of the immune responses in the patients, and for correlating these responses with the clinical outcomes of the infections and, possibly, immune protection. As secondary, IgG-dominated antibody responses depend on T cell help, knowledge of immunodominant serospecificities can lead to the specificities of anti-Leishmania T cells and to the identification of T cell epitopes, thus providing insights into the specificities of cellular immune responses. A number of antigens are known for VL. Most of them were initially described for infected or immunized animals and later tested with human sera. Only few were identified directly for patients. A general inventory of the range of the serospecificities in VL patients has not been attempted yet.

With this report we present a strategy for highly resolved mapping of serological specificities that allows to assess the range and specificities of immune responses to complex infectious agents such as protozoal parasites and, at the same time, to identify specific antigens. This strategy combines Western blot seroscreening with proteome technologies involving 2-dimensional polyacryl amid gel electrophoresis (2D-PAGE) and mass spectrometry (here, matrix assisted laser desorption/ionisation – time of flight mass spectrometry, MALDI-TOF MS) for the identification of the antigens.

## Results

### Antigenicity of Leishmania parasites in VL patients

As established by clinical diagnostics and a number of laboratory investigations, infection of an individual with LD induces vigorous serological and cellular immune responses [4], [5]. However, the scope of these responses in terms of the range of parasite antigens addressed and inter-individual variations has not been unraveled in detail. To gain detailed insights into the antigenicity of the parasites as detected by the serological specificities of VL patients, to assess the range of recurring and the degree of deviating specificities in the antibody responses, and to determine whether there is a hierarchy of antigens as to the frequencies and magnitudes of responses induced, we separated LD protein extracts by 1-dimensional gel electrophoresis and probed the respective Western blots with sera of clinically diagnosed VL patients. All the sera of VL patients were collected on the same day at the Kala-Azar Medical Research Center of the Banaras Hindu University located in the city of Muzaffarpur, district of Muzaffarpur in the Indian state of Bihar. Of the 15 patients included in this report (Table 1), most were from highly endemic foci around Muzaffarpur. The exceptions were patients number 1, 2 and 3 who were from Motihari in the district of Purbi Champaran, North-West of Muzaffarpur that is also highly endemic, and patient 6 who was from the mesoendemic district of Siwan in Western Bihar. Control sera were from 4 healthy housemate relatives of the patients. Ten of the patients were male, 5 female. Their average age was 25 years with a range of 7 to 45 years. They had been clinically diagnosed for VL on the day blood was drawn for serum production or up to 66 days before. The average time between diagnosis and blood retrieval for the study was 26 days. The time that had elapsed since the diagnosis of VL does not indicate the time of infection, which most likely had occurred months before, but is a rough indication of the time of obvious symptoms with high fever as indicator of vigorous immune responses against the infection. Seven patients were receiving sodium antimone gluconate for therapy, 8 had not been treated yet.

**Table 1:**
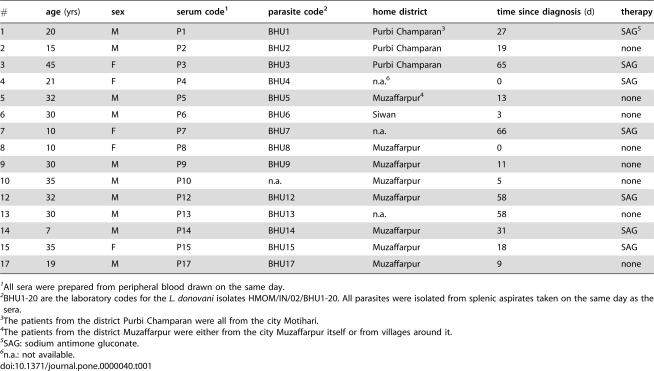
Patients, sera and parasites

#	age **(yrs)**	sex	serum code^1^	parasite code^2^	home district	time since diagnosis **(d)**	therapy
1	20	M	P1	BHU1	Purbi Champaran^3^	27	SAG^5^
2	15	M	P2	BHU2	Purbi Champaran	19	none
3	45	F	P3	BHU3	Purbi Champaran	65	SAG
4	21	F	P4	BHU4	n.a.^6^	0	SAG
5	32	M	P5	BHU5	Muzaffarpur^4^	13	none
6	30	M	P6	BHU6	Siwan	3	none
7	10	F	P7	BHU7	n.a.	66	SAG
8	10	F	P8	BHU8	Muzaffarpur	0	none
9	30	M	P9	BHU9	Muzaffarpur	11	none
10	35	M	P10	n.a.	Muzaffarpur	5	none
12	32	M	P12	BHU12	Muzaffarpur	58	SAG
13	30	M	P13	BHU13	n.a.	58	none
14	7	M	P14	BHU14	Muzaffarpur	31	SAG
15	35	F	P15	BHU15	Muzaffarpur	18	SAG
17	19	M	P17	BHU17	Muzaffarpur	9	none

1 All sera were prepared from peripheral blood drawn on the same day.

2 BHU1-20 are the laboratory codes for the *L. donovani* isolates HMOM/IN/02/BHU1-20. All parasites were isolated from splenic aspirates taken on the same day as the sera.

3 The patients from the district Purbi Champaran were all from the city Motihari.

4 The patients from the district Muzaffarpur were either from the city Muzaffarpur itself or from villages around it.

5 SAG: sodium antimone gluconate.

6 n.a.: not available.

**Figure 1:**
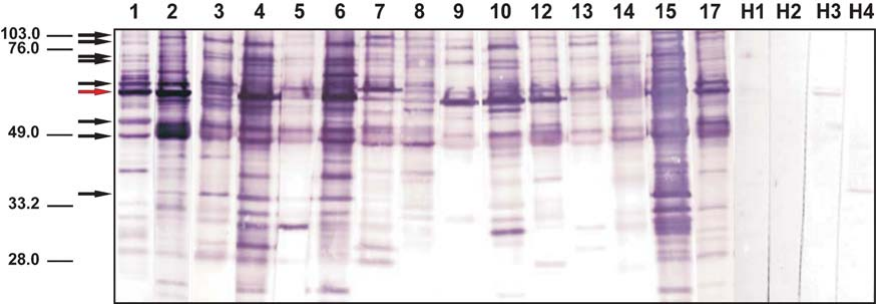
Crude extract of *Leishmania donovani* AG83 cells was separated in a 12% SDS-PAGE. After transfer of the protein onto nitro cellulose each lane was incubated separately with serum of 15 individuals with symptoms of VL (lanes 1–15) and 4 healthy donor controls (lanes 16–19).

The initial 1-dimensional Western blot analysis was done with the established LD laboratory strain AG83 as source of Leishmania antigens. AG83 had been isolated from a VL patient in West Bengal about 20 years earlier and did not display the possible antigenic variations of recent endemic parasites. The blots were developed with the same amount and dilution of serum in each case so that the different overall intensities of the signals reflect the different overall anti-LD serotiters, and the presence and intensities of the individual antigen bands the differences in the specificity patterns of the individual immune responses. The blots obtained with the 15 patient sera shown in Figure 1 reveals a broad range of immune specificities and extensive heterogeneity of the serological anti-LD responses in the individual patients. While several antigen bands occur in the blots with all sera, in no case such shared antigens are equally dominant. In fact, the pattern of dominant antigens reflected by the strong bands in the blots is different for every individual VL patient analyzed. Neither the overall intensities of the Western blot signals nor the individual antigenicity pattern correlate with the time since diagnosis, or age or sex of the patients. These analyses demonstrate that the antibody responses to the parasites are highly individualized, and that there are no uniformly dominant antigens and no recurring hierarchies of the antigens targeted by the immune responses of different patients.

### Patterns of the anti-Leishmania serospecificities in patients

**Figure 2:**
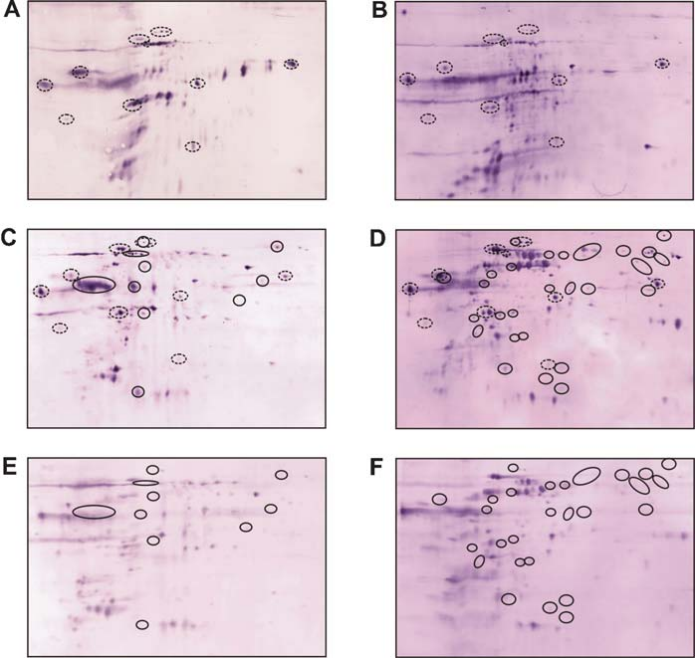
2D-Western blot analysis of the serospecificities of different VL patients and of the antigenicities of *L. donovani* isolates from different endemic districts in Bihar, India. Whole protein extracts of *L. donovani* BHU2 (MHOM/IN/02/BHU2) (Panels **A**–**D**) isolated from a patient from Motihari or BHU17 (MHOM/IN/02/BHU17) (Panels **E**–**F**) isolated from a patient from Muzaffarpur district were separated by 2-dimensional electrophoresis (pH-range of first dimension: 3–10 on 18 cm IPG-strips; second dimension: 12.5% SDS-PAGE). The separated proteins were blotted onto nitrocellulose membrane and probed with sera of different patients: **A**: patient No. 17; **B**: patient No. 2; **C**: patient No. 3; **D**: patient No. 14; **E**: patient No. 17; **F**: patient No. 3. Patients 2 and 3 were from Motihari, patients 14 and 17 from Muzaffarpur. For comparison of the serospecificities shown with Panels **A**–**D**, BHU2 proteins were probed with 4 different sera, 2 from Motihari, 2 from Muzaffarpur district. Dotted circles indicate antigens recognized by all 4 sera. Private specificities are not marked. For comparison of the antigenicities of different LD isolates shown with Panels **C**–**F**, proteins of BHU2 (Panels **C** and **D**) and BHU17 (Panels **E** and **F**) were probed with a serum from the same district and a serum from the respective other district. Circles with full lines in panels **C**–**F** indicate antigens that are detected only in one of the two LD isolates but not in the other.

The 1-dimensional Western blots from SDS-PAGE comprehensively document the antigenicity of the parasites and the range of the specificities of antibody responses against these parasites. The antigen bands detected in these analysis, however, are likely to include, depending on the specific patient serum, a varying number of different antigens. For highly resolved analyses of the antigenicity of LD and the specificities of the anti-parasite antibody responses in VL patients, we ran 2-dimensional Western blot analyses based on combinations of isoelectric focusing with a pH range of 3 to 10 and SDS-PAGE with parasites isolated from splenic aspirates of Patient 2 and probed the replica Western blots with autologous serum and sera of Patients 14, 17 or 3 (Figure 2A, B, C, D, respectively), whereby the parasites were from a patient from Motihari as were the sera p2 and p3, whereas the sera p14 and p17 were from patients from Muzaffarpur. Since the protein source is the same for all 4 blots shown, these analyses compare the specificities of the antibody responses of the 4 patients. The overall patterns of the spots are different in all 4 cases. Notwithstanding this heterogeneity, a large number of antigens are detected by all 4 sera. The most prominent of these shared antigens are circled with dotted lines in Figure 2A–D. The relative intensities of the signals in the different blots differ greatly indicating that the immunogenicity of the antigens differs in different patients. In addition to recurring specificities, an even larger number of antigens is detected by the sera of only 1 or up to 3 of the patient sera but not by the others. The range of intensities of the divergent signals is comparable to those of the shared ones. Some of the divergent spots are very intense in 1 or 2 blots but completely missing from the others. These differences are particularly noticeable in areas with clusters of antigens where some antigens of the clusters are detected by one serum but not by the other sera. A comparison of the specificity patterns of the sera of patients from the 2 different districts among each other and with the sera of patients from the respective other district reveals no correlation, again emphasizing the individuality of the serospecificities in responses to LD.

### Differential antigenicity of LD isolates

To analyze possible variations of the antigenicity of LD parasites we compared the 2-dimensional Western blot patterns of LD isolates from Motihari and Muzaffarpur probed with sera from the same or from the respective other district. This comparison is shown with panels C–F in Figure 2. Here, BHU2 is representative of Motihari LD isolates (Figure 2C and D) and BHU17 representative of Muzaffarpur isolates (Figure 2E and F). Both isolates were obtained from splenic aspirates collected on the same day. They represent the parasites of the same endemic season from endemic foci of two adjacent districts in Bihar, India. The sera used for the Western blots were from Patient 3 of Motihari (Figure 2D and F) and Patient 17 of Muzaffarpur (Figure 2C and E). Both sera produced similar antigenicity/specificity patterns when tested with the different parasite isolates (comparing Figure 2C with E, and D with F). These similarities of the specificity patterns with different isolates testifies to the reproducibility of the 2-dimensional electrophoresis. However, despite the overall similarities of the antigen patterns detected with the same serum, there are a number of differences which are circled in the panels Figure 2C–F where the corresponding areas of the antigen spots are pair-wise matched for the 2 different LD isolates. The intensities of the antigen spots vary and some of the weaker signals might just have dropped below detection limit in one of the panels that are compared. However, a number of very intense antigen spots detected in the Western blots with the BHU2 isolate are clearly missing from the Western blots with the BHU17 isolate. As it was the case for the blots with BHU2 parasites, the antigenicity pattern of BHU17 varies with the serum (comparing Figure 2E and F). Together, these comparisons expose differences in antigenicity of the 2 isolates. Both isolates have clearly been typed as *L. donovani*. In fact, all Leishmania parasites isolated from the endemic regions in the Ganges plane until now were *L. donovani* and were found to be of the same clade. None of the typing protocols applied to these isolates has ever demonstrated any species difference. Also the isolate used for the presented study had been typed and found to be of the same species (G. Schönian et al., to be published shortly). The different antigenicities seen in the 2D Western blots, thus, are antigenic variations in a closely related cluster within the same species. The differences are particularly noticeable in the case of the isolate BHU17 but also detected with isolate BHU2. With both sera, the reactivities were found to be weaker towards the LD antigens of the isolate obtained from the patients themselves than against the other isolate. This observation could be the result of selection processes by the immune system.

### Identification of Leishmania antigens in visceral leishmaniasis

**Figure 3:**
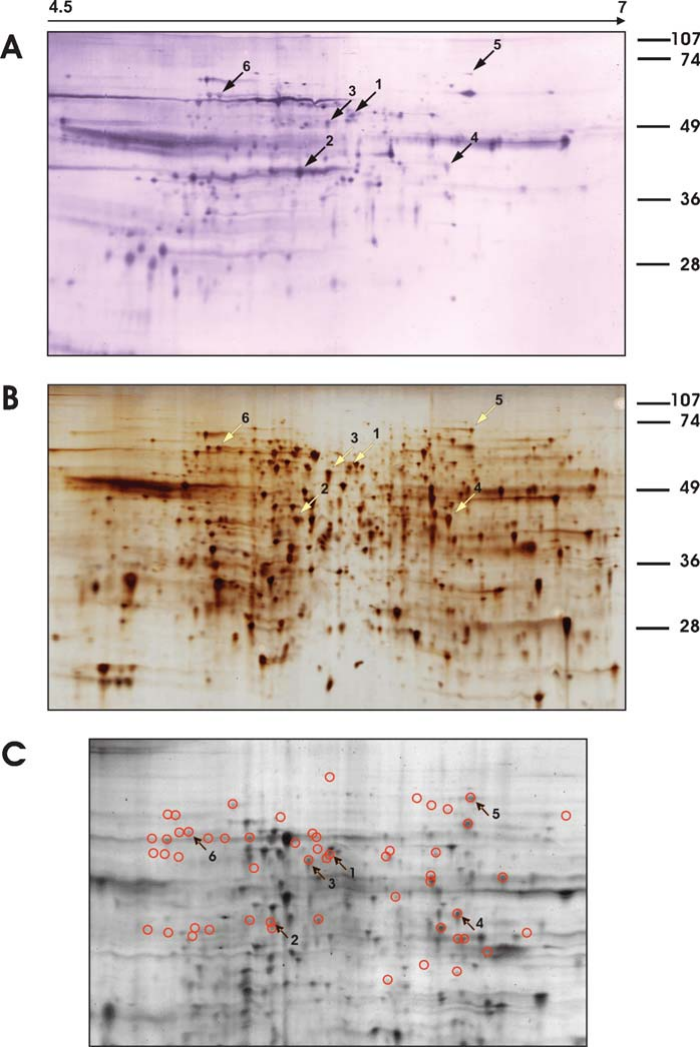
Mapping of the serospecificities of VL patients against *L. donovani* antigens. The proteins of total lysates of *L. donovani* isolate BHU2 (MHOM/IN/02/BHU2) promastigotes were separated by 2-dimensional gel electrophoresis with a pH gradient of 4.5–7 in the first dimension and a 12.5% SDS-PAGE in the second. **A**: Western blot developed with autologous serum. 330 Leishmania antigens could be counted. **B**: The corresponding silver-stained gel displaying 1067 protein spots. 68 of 330 antigens could be assigned to protein spots in the silver-stained gel. Six of the protein spots, indicated by arrows and numbered 1–6, were processed for protein identification by mass spectrometry. **C**: Inner section of a SyPro Ruby-stained gel with the same protein material. The proteins that match antigens detected by the Western blot analysis shown in Panel **A** are circled. 54 antigenic proteins are thus marked, 14 antigenic proteins are outside the sections shown. The arrows again indicate the six identified proteins.

The identification of antigens requires that the antigen spots of the Western blots are assigned to protein spots in silver-stained 2-dimensional electrophoresis gels. The high number of antigen spots allows to identify patterns that can be compared to the patterns obtained by protein staining and used to define the coordinates of the matching spots. Nonetheless, high densities of antigens and proteins in the two maps to be compared also raise the problem that small uncertainties in aligning individual spots might obscure proper assignment. Moreover, antigen detection by Western blotting is more sensitive than protein detection by silver staining. Antigens that produce intense signals in the Western blots may, therefore, well correspond to minor spots in the silver-stained gels. To enhance the resolution of the 2-dimensional separation and, thereby, improve the alignments of Western blots and silver-stained gels, we produced zoom gels with a pH range of 4.5 to 7, the region in the 2-dimensional gels that is most densely populated with protein spots. The proteome map obtained with the silver-stained zoom gels for parasite BHU2 and shown with Figure 3B displays 1067 clearly detectable protein spots, the corresponding 2-dimensional immunoblot obtained with the autogeneous serum some 330 antigen-spots (Figure 3A). It appears from the comparison of proteome map and immunoblot that a high fraction of the Leishmania proteome consists of antigens that induced specific responses in VL patient.

Of the 330 antigens 68 could unequivocally be assigned to protein spots in the silver-stained gels. In the remaining cases the match was uncertain. These results were reproduced in 9 independent silver-stained gels and 3 Western blots. Of the 68 matched antigens the 6 indicated by arrows in Figure 3 were identified from the silver-stained gel by mass spectrometry (see below). To compare antigenicity with the expression levels of the proteins we stained a 2-dimensional gel with the fluorescent dye SyPro Ruby which produces spot intensities that are proportional to the protein amount. Figure 3C shows a section of this gel with the antigens assigned to protein spots in the silver-stained gel circled. For orientation, the 6 identified antigens are indicated with arrows. A comparison of the Western blot in Figure 3A and the SyPro Ruby-stained gel in Figure 3C reveals that the antigenicity of the proteins does not correlate with their expression levels. There are a large number of strongly expressed proteins that are not or only very faintly detected as antigens in the Western blot and, vice versa, a number of very intense signals in the Western blot that correspond to very faint or even undetectable spots in the SyPro Ruby-stained gel.

**Figure 4:**
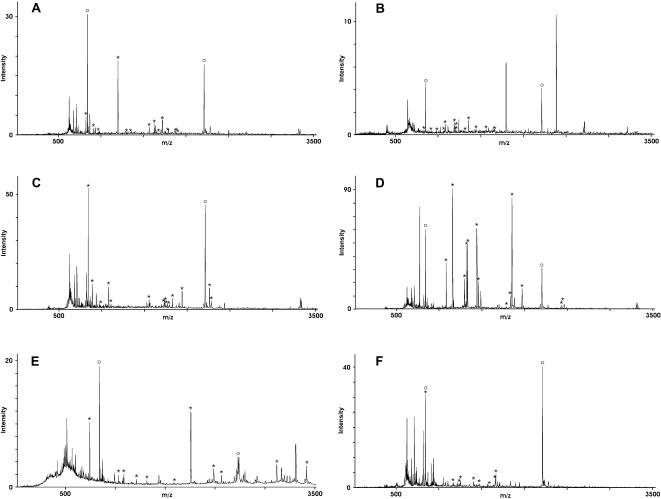
Identification of *L. donovani* antigens by mass spectrometry. The protein spots in the silver-stained gel that had been assigned to antigen spots in the corresponding Western blot were excised, destained and incubated with trypsin. The resulting fragments were extracted from the gel pieces and analyzed by MALDI-TOF-MS. Panels **A**–**F** show the peptide mass fingerprints (PMF) of the proteins in spots 1–6, respectively. Upon processing via MASCOT, the antigens were identified as HSP70 (spots 1, panel **A** and spot 2, panel **B**), gp63 (spot 3, panel **A**), EIF-4a (spot 4, panel **C**), Ef2 (spot 5, panel **D**) and grp78 (spot 6, panel **E**). The fragment masses that could be matched to theoretical trypsin digests of the identified proteins are indicated by asterisks. Open circles indicate the autolytic fragments of trypsin that were used for internal calibration.

**Figure 5:**
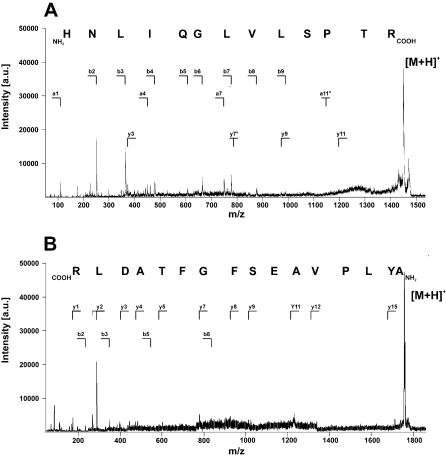
Confirmation of the protein identifications for spots 4 and 5 by PSD mass spectrometry for peptide fragmentation fingerprint (PFF) analyses. Panel **A** shows the PSD spectrum of a 1447 Da fragment of the protein in spot 4 and panel **B** that of a 1757 Da fragment from antigen spot 5. The former was identified as the tryptic fragment HNLIQGLVLSPTR from EIF-4a corresponding to the *L. major* sequence of this protein and the latter as the tryptic fragment AYLPVAESFGFTADLR of EF2 also of *L. major*. In both cases, the protein identification by PFM analyses were, thus, confirmed by PFF analyses and homology to the sequences of the proteins in *L. major*.

For identification, the six protein spots were excised from the silver-stained gel and the proteins fragmented in the gels with trypsin. The tryptic fragments were extracted and analyzed by mass spectrometry. The resulting mass pattern of these fragments (peptide mass fingerprints, PMF) are shown in Figure 4 for the 6 antigens. These patterns were used for MASCOT database searches for corresponding PMF pattern generated from the sequence database entries. The searches identified the antigens as HSP70 (spots 1 and 2), gp63 (spot 3), the initiations factor EIF-4A (spot 4), the elongation factor EF2 (spot 5) and grp78 (spot 6). The percentages of matched masses and sequence coverage are listed for each protein in Table 2. Remaining masses that could not be assigned to the identified protein were extensively reanalyzed but did not produce any evidence for proteins than the identified ones which supports the identification of the antigens. Table 2 lists also the theoretical masses and isoelectric points calculated for the proteins from their sequences, and the corresponding experimental values deduced from the 2-dimensionen electrophoresis gel. The identifications of the proteins were of sufficiently high significance for HSP70, gp63 and grp78. To confirm the results for EIF-4A and EF2, one of the matched fragments each was subjected to post-source decay (PSD) analysis. The PSD spectra for these 2 fragments, the assignments of the masses to the N- and C-terminal fragment series and the interpretation of these signals in terms of amino acid sequence are provided with Figure 5A and B. In both cases the resulting fragment sequences, HNLIQGLVLSPTR for the spot 4 fragment (Figure 4C and 5A) and AYLPVAESFGFTADLR for the spot 5 fragment (Figure 4D and 5B) match exactly the sequences of the proteins identified by PMF analysis, i.e. EIF-4A and EF2, respectively. For the antigens in spots 1, 4, 5 and 6 the theoretical and experimental values are in good agreement. The theoretical and experimental masses of antigen spot 3 also agree very well with the sequence of the identified protein gp63. The difference of about 1.4 pH units in the pI values can be explained by the fact that gp63 is an outer-membrane glycoprotein rich in sialic acids that confer additional negative charges to the protein. The antigen in spot 2 was identified, like spot 1, as HSP70. While its isoelectric point corresponds well to the expected value, its mass is with 45 kDa more than 20 kDa too small. For both spots, the identifications are clear and 6 of the tryptic fragments are the same for both proteins. An obvious explanation for the discrepancy would be that the protein in spot 2 is a truncated version of the HSP70 found in spot 1. However, this explanation is excluded by the fact that the tryptic fragments assigned to the protein sequences scatter along the entire extend of the respective proteins, between sequence position 73 and 610 for spot 1 and 51 and 610 for spot 2. The alignment of the tryptic fragments of the spot 2 protein with the sequence of HSP70 shows a stretch of 210 amino acids corresponding to about 25 kDa without a matching tryptic fragment. For spot 1 there are 2 fragments within this region. Alternative explanations for the low mass of the protein in spot 2 could be that it is a homologue of HSP70, or product of trans- or kinetoplastid-specific cis-RNA splicing mechanisms commonly found in kinetoplastid protozoa such as Leishmania [22] but also described for other species and phyla [23]. In fact, we have found substantial heterogeneity in the 3′ part of HSP70 mRNA from LD including one clear case of trans-splicing [24]. These observations do not prove but would fit the second explanation for the deviating mass of the spot 2 antigen. Seroreactivity of the p2 serum towards HSP70 had also been demonstrated with a recombinant bacterial expression clone for the full length gene product [24], thus, confirming the identification of this antigen.

**Table 2:**
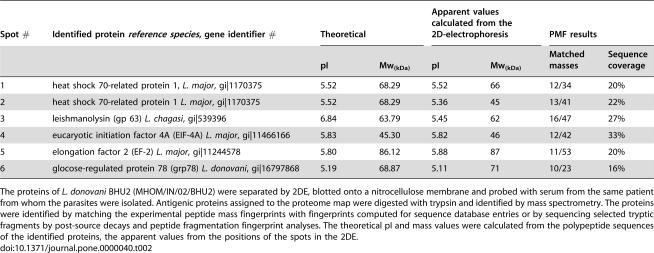
Antigens in human visceral leishmaniasis identified by proteome serology.

Spot #	Identified protein *reference species*, gene identifier #	Theoretical		Apparent values calculated from the 2D-electrophoresis		PMF results	
		pI	Mw_(kDa)_	pI	Mw_(kDa)_	Matched masses	Sequence coverage
1	heat shock 70-related protein 1, *L. major*, gi|1170375	5.52	68.29	5.52	66	12/34	20%
2	heat shock 70-related protein 1 *L. major*, gi|1170375	5.52	68.29	5.36	45	13/41	22%
3	leishmanolysin (gp 63) *L. chagasi*, gi|539396	6.84	63.79	5.45	62	16/47	27%
4	eucaryotic initiation factor 4A (EIF-4A) *L. major*, gi|11466166	5.83	45.30	5.82	46	12/42	33%
5	elongation factor 2 (EF-2) *L. major*, gi|11244578	5.80	86.12	5.88	87	11/53	20%
6	glocose-regulated protein 78 (grp78) *L. donovani*, gi|16797868	5.19	68.87	5.11	71	10/23	16%

The proteins of *L. donovani* BHU2 (MHOM/IN/02/BHU2) were separated by 2DE, blotted onto a nitrocellulose membrane and probed with serum from the same patient from whom the parasites were isolated. Antigenic proteins assigned to the proteome map were digested with trypsin and identified by mass spectrometry. The proteins were identified by matching the experimental peptide mass fingerprints with fingerprints computed for sequence database entries or by sequencing selected tryptic fragments by post-source decays and peptide fragmentation fingerprint analyses. The theoretical pI and mass values were calculated from the polypeptide sequences of the identified proteins, the apparent values from the positions of the spots in the 2DE.

The experiments for the assignment and identification of the antigens was done 4 times, once with a silver-stained gel (shown in the Figure 3), once with a Coomassie-stained gel and twice with SyProRuby-stained gels (one example shown in Figure 3). Five of the antigens were identified in all 4 gels, one, EF2 (spot 5) 3-times, i.e. in the silver- and the two SyPro Ruby-stained gels, not in the Coomassie-stained gel which is most likely due to the lower sensitivity of Coomassie staining compared to the other two techniques. To test the reproducibility of the spot pattern in the protein-stained gels and Western blots, all together, 13 gels were run with BHU2 proteins, 2 stained with SyPro Ruby, 2 with Coomassie and 9 with silver. 4 Western blots were run. The reproducibility of the spot pattern in the gels and blots was very high and the 68 antigens could be assigned to the silver-stained gels in nearly all cases.

## Discussion

The proteome-serological approach described here produces comprehensive and highly resolved representations of the antigenicity of *L. donovani* parasites and the specificities of anti-Leishmania immune responses in visceral leishmaniasis patients that is beyond the capabilities of previous attempts and technologies. The numbers of antigens detected in 2-dimensional immunoblots for individual patients exceed 330, a number that corresponds to more than 30% of the number of protein spots detectable in silver-stained electrophoresis gels for the same parasites. Although these figures are far higher than any earlier analyses have exposed, they still are likely to be low estimates of the real complexity of the antibody responses against the parasites in human leishmaniasis. A comparison of the specificity patterns of sera from different patients reveals great differences in the antigens targeted. No two VL patients develop antibody responses with even closely similar profiles. Nonetheless, there is a large number of shared specificities that can be instrumental in the development of diagnostics or vaccines. But also these shared specificities are represented in the different patients with different intensities and hierarchies. There is no immunodominant antigen or set of antigens, there is not even an antigen that dominates the immune responses in a larger fraction of the patients. This holds true also when the specificity patterns are compared within age and sex groups or related to severity or the time since onset of the disease. However, there still may be specificity patterns that are indicative of immunity and protection against reinfection, or the likelihood of cutaneous leishmaniasis, Post-Kala Azar Dermal Leishmaniasis (PKDL), after initial VL [4]. As demonstrated by the data presented here, diagnostic arrays based on seroresponses to the sets of antigens that most often are targeted are expected to be far more sensitive and indicative of the outcome of the disease than simple monospecific diagnostics such as the currently widely applied rK39 dipstick [6], [25], [26]. It will be up to long-term proteome-serological follow-up studies to identify such patterns, in particular, with individuals who are seropositive but disease-free and patients who develop PKDL versus those who do not. The identification of specificity patterns might also be guidance for the search for candidate vaccine antigen. Currently, for development of vaccines with defined antigenicity there is a focus on one or few antigens such as LACK, LeIF, TSA, gp63 and LmSTI-1 (27, www.who.int/vaccine_research/). Although some of these were confirmed with the present study as relevant antigens in human visceral leishmaniasis, it might be advisable to define sets of antigens that correlate with protection, and to design vaccines that induce broad and complex immune responses. As it becomes increasingly clear that T cells are crucial for effective anti-Leishmania immune responses, the composition of the vaccines should be designed to address these cells as well. While there are varying factors that could be responsible for the enormous heterogeneity of the immune responses in VL patients, the immunogenetics of the patients, i.e. HLA control of T cell responses, is expected to have a major part in this. Future studies will have to correlate the specificities of serological and cellular immune responses with HLA genetics, and with individual courses of the disease and development of immunity as exemplified by seropositive but disease-free individuals.

In addition to the specificities of the anti-Leishmania immune responses, the proteome serological analyses also show differences in the antigenicity of the parasites, in particular, when the seroresponses against parasites from different but neighboring endemic areas in Bihar are compared. Most striking are losses in antigenicity seen when the seroreactivities of individual patients against parasites isolated from their own splenic aspirates are compared to those against parasites isolated from patients of a distant endemic area. Since the parasites in all cases are LD of the same clade, these differences seem to indicate antigenicity drifts in the parasites. They do not correlate with major differences in the proteomes of the different parasite population as detectable in silver-stained 2-dimensional electropherograms. Loss of antigenicity as indicated by these data suggests immune evasion by the parasites. Such responses to the hosts immune responses have been described for other protozoans such as plasmodia [28], [29] and trypanosomes [30]–[33], and related primarily to the persistence of the infections in the individual host [34]–[36]. Long-term persistence in a host is essential for the parasites as they have to shelter and propagate for a sufficiently long time to have a chance to be taken up by a sand fly vector again for re-starting their life cycle and transmitting their progenies to new hosts. This need to escape immune attacks for an extended period is particularly important in the endemic areas in India where *Leishmania donovani* infections are anthroponotic, i.e. without extrahuman vertebrate host, and endemic outbreaks are spaced by extended periods of low-prevalence [37], [38].

The data presented here provide new insights into the quality and identity of the antigens targeted by the immune systems of leishmaniasis patients. Requena and colleagues had suggested that anti-Leishmania antibody responses are preliminarily directed against highly expressed conserved antigens that are typically parts of multi-component complexes such as heat-shock proteins, ribosomal proteins or proteins of the DNA replication and the transcription machineries. These antigens were dubbed panantigens [39], [40]. While the antigens we identified fit this category of proteins [41]–[45], the high numbers of antigens, many of them low abundance proteins, would rather testify against such generalization and suggest that, due to technical shortcomings such as sensitivity of the analytical procedures for protein identification, highly expressed proteins are more likely to be identified than highly antigenic but poorly expressed proteins. The number of antigens detected, the high antigenic coverage of the proteome of the parasite and the great variation in the specificity profiles seen with sera from different patients strongly suggest that many different categories of proteins can be antigens.

As illustrated with the results presented herein, proteome serology is a powerful and highly resolving technology that provides an overall inventory of the antigenicity of the infectious agents and of the specificity pattern of the immune responses against complex pathogens. At the same time it allows to identify defined antigens as well as antigenic patterns which may advance the development of differential diagnostics and of vaccines. Notwithstanding these potentials, proteome serology is also a demanding technology with a number of difficult steps [46]. Problems may arise when protein-stained gels are compared with Western blots to assign antigen to protein spots for subsequent identification. With large numbers and high densities of proteins and antigens, proper assignment can be difficult. In parts, this problem is due to the differences in sensitivity of Western blot and protein detections. In the work presented herein, we employed a series of measures to define the coordinates of the antigens in the protein-stained gels exactly (see Materials and Methods). Although pattern recognition software may facilitate the task of assigning antigen and protein spots, manual editing of the results appears inevitable. In addition, the identification of the proteins by peptide mass fingerprint or peptide fragmentation fingerprint analyses is easily obscured by a few amino acids exchanges in the protein sequences. The *L. donovani* genome has not yet been sequenced so that the present work had to be done with information of the *L. major* genome sequence database and occasional *L. donovani* protein sequences. Differences in protein sequences between these two Leishmania species can be responsible for some of the failures to identify the antigens. In cases where the amounts of proteins in the gels permit, the limitation of the usual peptide mass or peptide fragmentation fingerprint analyses for protein identification in proteomics can be overcome by *de novo* sequencing of tryptic fragments [47]. Also, the rapidly progressing genome project for *L. infantum* which is closely related to *L. donovani* will improve the success rates in protein identification from 2-dimensional electrophoreses. In any case, sensitivity remains a problem. Since prominent antigens are not necessarily highly expressed proteins, their identification often fails due to lack of sufficient amounts of material for mass-spectrometric identification. Despite these difficulties, proteome serology appears to be superior to other approaches to antigen discovery and will yield substantial new information that will be instrumental in the development of new diagnostics and vaccines.

## Materials and Methods

### Parasites and sera

LD used are MHOM/IN/02/BHU2 (BHU2), MHOM/IN/02/BHU17 (BHU17) and AG83. BHU2 and BHU17 are recent isolates from spleen aspirates of VL patients from highly endemic foci in Bihar, India [24]. The time courses of growth of the isolates and lines used in the study were established and all parasites harvested for analyses from the same time point of late-log growth phase of early-passage cultures. The parasites were cultured as promastigotes in medium M199 (Gibco BRL, Heidelberg) with 20% FCS (Biochrom AG, Berlin), 20 mM HEPES, 4 mM sodiumbicarbonate and 20 U/ml penicillin/streptomycin at 22°C. The sera used for Western blot analysis were from 15 Indian VL patients (P1–10, 12–15 and 17) and, as controls, 4 healthy housemate relatives (H1–H4) of patients. The numbering of the patient sera matches the numbering of the parasite isolates, thus, sera P2 and P17 were from the same patients as the parasite isolates BHU2 and BHU17. The study had been reviewed and approved by the institutional ethics committees of the Kala Azar Medical Research Center, Banaras Hindu University, Varanasi, the Indian Institute of Chemical Biology (IICB), Calcutta and the Charité – Universitätsmedizin Berlin as well as by the Indian national ethics committee at the Indian Council of Medical Research (ICMR) and the Indian Health Ministry's Screening Committee.

### Protein sample preparation

The parasites were harvested by centrifugation at 2,050 × g for 30 minutes at 20°C, washed thrice with PBS and solubilized in lysis buffer (7 M urea, 2 M thiourea, 2.5% Triton X-100, 2% β-mercaptoethanol, 0.8% Pharmalyte pH 3.5–10 (LKB, Freiburg, Germany), 200 µM Pefablock® (Merck, Darmstadt), 1 µM pepstatin (SIGMA, Munich, Germany) and 10 µM leupeptin (SIGMA, Munich, Germany) as adapted from Görg and Chan [48], [49] by vortexing and sonicating for 10 minutes in an ice cooled waterbath. The cell extracts were then incubated for one hour at room temperature (RT) with 4,000 U/ml benzonase (Merck, Darmstadt, Germany) to degrade nucleic acids and then centrifuged at 350,000 × g for 15 minutes at 15°C. The supernatants were collected, incubated one more time with Benzonase for 10 minutes at RT and cleared by ultracentrifugation as before.

### Two-dimensional polyacrylamid gel electrophoresis

Isoelectric focussing (IEF) was done in immobilized pH-gradient gel strips (IPG strips 180 mm × 3 mm; Pharmacia, Freiburg) with pH ranges of 4 to 7 or 3 to 10 [48], [49]. Approximately 100 µg protein, extract of 1×10^8^ cells, in 350 µl solubilization buffer were applied per IPG strip. The samples were loaded overnight at RT by in-gel re-swelling under silicon oil in a nitrogen and water saturated atmosphere to prevent oxidation of the protein and drying of the gel strips. The loaded IPG strips were rinsed, mounted on a cooled ceramic plate and connected with the electrodes via water-wetted paper bridges to the electrode buffers. The IEF was run at 20°C under silicon oil in a nitrogen- and water-saturated atmosphere. The electric parameter were 0.15 mA per IPG strip and voltage settings stepwise increased 18 h 50 V, 1 h 150 V, 2 h 300 V, 1 h 600 V, 24 h 3,500 V and 3 h 5,000 V for a total of 101,250 Vh for pH 4–7 IPG strips and 18 h 50 V, 1 h 150 V, 2 h 300 V, 1 h 600 V, 6.5 h 3,500 V and 3 h 5,000 V for a total of 40,000 Vh for pH 3–10 IPG strips. After the run, the IPG-strips were stored at −20°C. For the second dimension, the IPG strips were thawed, rinsed with ultrapure water and equilibrated to SDS-PAGE conditions for 15 minutes in equilibration buffer (6 M urea, 30% glycerol, 2% sodium dodecylsulfate (SDS), Tris pH 6.8 and bromophenol blue, 1% dithiothreitol (DTT)) followed by 15 minutes in the same buffer but with 4% iodoacetamide instead of DTT. The equilibrated IPG-strips were rinsed with de-ionized water and placed gel-side to gel-side onto the 4.8% acryl amide, 0.13% bisacryl amide stacking gel of a horizontal SDS polyacrylamide gel with a 12.3% acryl amide, 0.34% bisacryl amide separation gel. The settings for the runs were 1,000 V, 40 W and 20 mA for 2–3 h for the pre-run to transfer the protein from the IPG strip into the SDS polyacrylamid gel, followed by 1,000 V, 40 W and 40 mA for the separation until the running front reached the anodic end of the gel.

### Protein staining

The gels were stained with the highly sensitive silver staining approach according to Blum and colleagues [50]. Briefly, SDS-PAGE gels were fixed in a solution of 40% Methanol and 10% acetic acid for one hour or overnight. Then, the gels were washed thrice for 20 minutes in ultrapure water, sensitized for one minute in 0.02% sodium thiosulfate, washed three times for 20 seconds in water and incubated for 20 minutes in silver-staining solution (0.2% silver nitrate, 0.0074% formaldehyde). After washing thrice for 20 seconds in water, the gels were incubated in developing solution (6% sodium carbonate, 0.00015% formaldehyde, 0.0004% sodium thiosulfate) until protein spots are visible. The reactions were stopped with 0.025% EDTA in water. SyPro Ruby (Invitrogen, Heidelberg, Germany) staining was done according to the manufacturer's instructions.

### Western blot

The proteins from unstained SDS-PAGE were transferred onto nitrocellulose membranes (Schleicher & Schüll, Dassel, Germany) by semi-dry blotting for 2 hours at 400 mA. Free binding sites on the membranes were blocked with 5% low fat milk powder in Tris-buffered saline (TBS) for one hour at room temperature or at 4°C overnight. After blocking, the membranes were incubated with patient sera at a 2,000-fold dilution for one hour at room temperature, washed thrice for 10 minutes with TBS and incubated for 30 min with alkaline phosphatase-labelled anti human IgG (Anti-Human Ig-AP, Fab fragment; Boehringer Mannheim) at a 5,000-fold dilution. After washing three times for 10 minutes with TBS, the membranes were equilibrated to developing buffer (100 mM NaCl, 100 mM Tris-HCl, pH 9.5) and developed in the dark with 100 µl BCIP and 100 µl NBT in 100 ml developing buffer until antigen spots are visible. The reactions were stopped by replacing the developing solution with water. For 1-dimensional Western blots, the parasite proteins were separated by SDS-PAGE, 12% acryl amide, 0.8% bis-acryl amide, and blotted 1 h with 60 mA and processed as above.

### Matching antigens and protein spots

To match antigen spots in Western blots with the corresponding protein spot in the silver-stained gel, the coordinates of the blots and the gel were defined, first, with artificial spots at the corner points, Ponceau S staining of the blot filter and aligning the spot pattern with the spot pattern of the silver-stained gel and partial blotting of a master gel and definition of marker spot sets, second, definition of spot pattern in the local environments of the antigen spots to match blots and gels in these local regions accurately, third, comparing the sizes and shapes of the antigen and protein spots and considering only those that are alike in these two parameter.

### In-gel digestion of protein

The protein spots were excised manually with a self-made spot picker and de-stained as described by Gharadaghi and colleagues [51] with 50 µl of Farmer's reducing solution (15 mM potassium ferricyanide and 50 mM sodium thiosulfate, both dissolved in water) and then washed three times for 5–10 minutes with 150 µl water. Afterwards the gel spots were soaked in acetonitrile and dried under vacuum. The gel pieces were re-swollen in 7.5 µl of 5 mM ammonium bicarbonate with 75 ng of modified porcine trypsin (sequencing grade, modified; Promega; Madison, USA) to fragment the protein. After 10 minutes, 7.5 µl of 5 mM ammonium bicarbonate were added and the solution with the gel pieces incubated for at least 4 hours in a 37°C water bath. For MS analysis, 1.5 µl of the aqueous supernatants were mixed with 1 µl of 2,5-dihydroxybenzoic acid (DHB) (SIGMA, Munich, Germany) (5 mg/ml water) directly on MALDI targets (MTP AnchorChip 600/384, Bruker Daltonik, Bremen) and air-dried.

### Mass spectrometry

The mass spectrometry (MS) was done with a Reflex IV MALDI-TOF mass spectrometer (MS; Bruker Daltonik, Bremen) in reflection mode at an acceleration voltage of 20 kV. The MS was calibrated either with angiotensin II (1046.5 Da), angiotensin I (1296.6 Da), bombesin (1619.8 Da), substance P (1347.7 Da), ACTH 1–17 (2093.0 Da) and ACTH 18–39 (2465.1 Da) as external standards and with the autolytic 842.50 Da and 2211.10 Da trypsin fragments as internal standards. Monoisotopic peptide masses were measured. The spectra were processed by the “Xmass” software (Bruker Daltonik, Bremen) and the peaks annotated manually. Post-source decay (PSD) analyses were done in 12 sections for the entire mass range and data accumulated with up to 300 shots per section.

### Database analyses

The peak lists of the mass spectra were used for peptide mass fingerprint analyses with the Mascot software (Matrix Science; http:www.matrixscience.com/search_form_select.html) and profound (prowl; http://prowl.rockefeller.edu/profound_bin/WebProFound.exe) together with the NCBI sequence database. Most proteins were identified using the following parameter: database: eukaryota (eucaryotes); enzyme: trypsin; variable modifications: oxidation (M), propionamide (C); mass values: monoisotopic; protein mass: unrestricted; peptide mass tolerance: ± 100–200 ppm; peptide charge state: 1+; maximum missed cleavages: 1. The analyses of the PSD datasets were done either by peptide mass fingerprint or peptide fragmentation fingerprint analysis with Mascot.
